# Integrated transcriptome and metabolome analyses reveals the mechanisms of function loss of *Lr29* leaf rust resistance gene at high temperatures in wheat

**DOI:** 10.3389/fpls.2025.1537921

**Published:** 2025-02-26

**Authors:** Liwen Wang, Yang Yu, Hang Li, Mingzhu Lu, Shubo Cao, Ziqi Li, Haoyuan Song, Laszlo Purnhauser, Jinlong Li, Jiajie Wu

**Affiliations:** ^1^ State Key Laboratory of Wheat Improvement, College of Agronomy, Shandong Agricultural University, Tai’an, China; ^2^ Laboratory of Plant Pathology, Cereal Research Non-Profit Co. Ltd., Szeged, Hungary

**Keywords:** differentially expressed genes, *Lr29* gene, metabolome, *Puccinia triticina*, temperatures, transcriptome

## Abstract

Leaf rust (LR) is one of the most common diseases of wheat. The resistance gene *Lr29* provides wide resistance to LR, but loses its function under high temperatures. Despite the importance of this gene, the mechanism of resistance is unclear. In this study we investigated the resistance mechanism of the *Lr29* gene to LR at the seedling stage, as well as the reasons behind the loss of gene function at high temperatures by using integrated transcriptome and metabolome analyses. Results suggests that the pathways of reactive oxygen species (ROS), which could be due to expression of genes including LOX (lipoxygenase), APX (ascorbate peroxidase) and GST (glutathione S-transferase), play a key role in the resistance of *Lr29* to LR, furthermore flavonoids, such as epicatechin, cosmosiin, apiin, vitexin and rutin, were identified as the key metabolites linked to *Lr29* resistance. We also found that, at high temperatures, *Lr29* downregulated the genes and metabolites associated with glycolysis and the tricarboxylic acid (TCA) cycle, while genes and metabolites related to the shikimic acid pathway were upregulated. This study might provide a valuable theoretical foundation for the cloning of the *Lr29* gene, the analysis of its disease resistance mechanism, and the understanding of how temperature affects gene function.

## 1 Introduction

LR of wheat, caused by *Puccinia triticina* Erikss. (*Pt*), is one of the most prevalent and severe diseases of wheat globally. In temperate zones, yield losses due to LR typically range from 5% to 20%, but can reach to 50% during severe epidemics (Eversmeyer and Kramer, 2000). The extent of yield loss varies depending on factors such as weather conditions (especially, temperature and air humidity), timing of infection, and host resistance. Furthermore, climate change has accelerated the onset of LR, posing additional challenges to wheat production.

To date, over 80 LR resistance genes have been catalogued in wheat (Ren et al., 2023). Most of them confer race-specific resistance, and effective in all wheat growth stage (all-stage resistance, ASR), which can be easily identified in seedling stage (seedling resistance). ASR is shown by hypersensitive reactions around the sites of infection (McIntosh et al., 1995). Some of these genes like *Lr29* still confers strong resistance to *Pt* pathogens globally (Huerta-Espino et al., 2011). However, the *Lr29* gene is classified as a low-temperature resistance gene, which is sensitive to temperature fluctuations and exhibits a high infection rate when temperatures rise during the seedling stage. Additionally, rising temperature has been shown to cause the *Lr29* gene to lose its disease resistance during the seedling stage (Chen and Qin, 2002).

LR resistance gene *Lr29* is originated from chromosome 7E of *Thinopyrum elongatum* which was transferred to chromosome 7DS of common wheat. The ‘Thatcher’ base near-isogenic line carrying *Lr29* (Lr29NIL) was developed by crossing wheat with *Th. elongatum* and subsequently incorporating it into the genetic background of cv. ‘Thatcher’ through a series of backcrosses (Sears, 1977; Dyck and Lukow, 1988). Cytological observations of ‘Lr29NIL’ confirmed the translocation of the short arm and part of the long arm of the 7E chromosome from *Th. elongatum* to the 7D chromosome of wheat. The short arm of chromosome 7E contains the *Lr29* gene, and this translocation occurs near the centromere end of chromosome 7DL (Friebe et al., 1996).

Multi-omics technologies have emerged as powerful tools for studying plant systems, incorporating data from genomics, transcriptomics, proteomics and metabolomics. As gene expression changes over time in response to different stimuli, transcriptome profiling has demonstrated significant potential for analyzing gene expression. Metabolomics, on the other hand, offers valuable insight into plant physiology by examining various metabolites involved in diverse cellular processes (Yang et al., 2021). Combining transcriptome and metabolome analyses provides an effective approach for investigating plant disease resistance mechanisms.

In recent years, multiple studies on wheat yellow (Chen et al., 2013; Davoudnia et al., 2024; Lv et al., 2024; Nazarov et al., 2024) stem (Vishwakarma et al., 2023) and leaf (Hulbert et al., 2007) rusts molecular mechanism have been done, however, for the mechanism of *Lr29* gene and especially it’s sensitivity to high temperature, such studies are lacking. The objective of present study was to investigate the resistance mechanism of the *Lr29* gene to LR at the seedling stage, as well as the reasons behind the loss of gene function at high temperatures by using integrated transcriptome and metabolome analyses.

## 2 Materials and methods

### 2.1 Plants material and LR inoculation with *Pt*

In this study, the ‘Thatcher’ based ‘Lr29NIL’ carrying *Lr29* gene was used as LR resistance material, while cv. ‘Thatcher’ itself served as susceptible control. The ‘Lr29NIL’ was provided by Dr. Laszlo Purnhauser from the Cereal Research Company, Szeged, Hungary. The wheat *Pt* race D-3-2-182 virulent to cv. ‘Thatcher’ and avirulent to ‘Lr29NIL’ was provided by Dr. Shisheng Chen from the Institute of Advanced Agricultural Sciences, Peking University, China. Plants were scored at ~10 days post infection (dpi) using a 0 to 4 infection types (IT) scale (Stakman et al., 1962), where 0 as well as 1 IT scores represent resistance, 2 IT score – moderate resistance, 3 IT score – moderate susceptibility, 4 IT score – susceptibility. The plants were grown under a photoperiod of 16 h of light at 20°C and 8 h of darkness at 15°C. Seedling leaves were inoculated with spores mixed with talc powder (or *Lycopodium* powder), and the inoculated seedlings were kept in darkness for 24h at 20°C and 100% relative humidity. After this period, half of the seedlings transferred to a low temperature environment (16 h of light at 20°C and 8 h of darkness at 15°C), while the other half part was placed to a high temperature environment in a different incubator (16 h of light at 30°C and 8 h of darkness at 25°C). Low temperature and high temperature treatment of the sample are denoted by “L” (LLr29NIL vs LThatcher) and “H” (HLr29NIL and HThatcher), respectively.

RNA samples were collected at 0, 24, and 72 hours post infection (hpi), with three independent biological replicates for each time point. Metabolomes were sequenced at 72 hpi, with six independent biological replicates for each sample. The samples were rapidly frozen in liquid nitrogen and stored at -80°C.

### 2.2 RNA extraction, quality control, and RNA-sequencing

Total RNA was isolated with TRIzol reagent (Takara, Dalian, China) following the manufacturer’s instructions. Sequencing libraries were prepared with the NEBNext^®^ Ultra™ RNA Library Prep Kit for Illumina (NEB, USA), incorporating index codes to assign sequences to individual samples. The library fragments were purified using the AMPure XP system (BeckmanCoulter, Beverly, United States) to select cDNA fragments ranging from 150 to 200 bp in length. Purified PCR products were further cleaned using the AMPure XP system, and library quality was assessed using the Agilent Bioanalyzer 2100 (Agilent Technologies, Palo Alto, Calif.).

Following the manufacturer’s guidelines, the index coded samples were clustered with the cBot Cluster Generation System using the TruSeq PE Cluster Kit 3-cBot-HS (Illumina). After cluster generation, the library preparations were sequenced on the Illumina platform, yielding in 150-bp paired-end reads.

### 2.3 Transcriptome data analysis

The reads from each of the ‘Lr29NIL’ were mapped to Tel genome (*Th. elongatum* v1.0) and the wheat genome (‘IWGSC CS v2.1’), respectively (Appels et al., 2018; Wang et al., 2020). Reads from the ‘Thatcher’ samples were mapped to the wheat genome (‘IWGSC CS v2.1’) using TopHat (version 2.0.12) (Kim et al., 2013). To quantify the number of reads mapped to each gene, we utilized HTSeq version 0.6.1 (Anders et al., 2015). FPKM (Fragments Per Kilobase of exon model per Million fragments mapped) was then calculated as a measure of gene expression. To estimate the expression levels of all transcripts and assess mRNA abundance, we employed StringTie and ballgown (http://www.bioconductor.org/packages/release/bioc/html/ballgown.html) (Pertea et al., 2015; Kovaka et al., 2019). The differential expression of genes between the two groups was analyzed using DESeq2 software (version 1.22.1) (Robinson et al., 2010; Love et al., 2014). Genes with a *p*-value < 0.05 and absolute fold changes > 1.5 were classified as differentially expressed genes. These differentially expressed genes (DEGs) were subsequently enriched for GO (Gene Ontology) functions and KEGG (Kyoto Encyclopedia of Genes and Genomes) pathways (Subramanian et al., 2005; Kanehisa et al., 2021).

### 2.4 Metabolite extraction and metabolic profiling analysis

We conducted non-targeted metabolic profiling using liquid chromatography-tandem mass spectrometry (LC-MS/MS) to investigate plant metabolic changes following infection and elevated temperatures. Samples at 72 hpi, six replicates of both ‘Lr29NIL’ and ‘Thatcher’ were collected at different temperatures and then were ground in liquid nitrogen. To each 100 mg frozen powdered samples, 1 mL of extracting solution (methanol: acetonitrile: water 2:2:1) was added. The samples underwent ultrasound treatment at room temperature for 20 minutes, followed by centrifugation at 4°C and 13,000 rpm for 5 minutes. The supernatant was then transferred to a new centrifuge tube and evaporated using a rotary evaporator. The dried metabolite pellets were redissolved in 100 μL of 50% methanol, filtered through a 0.22 μm membrane, and analyzed using UPLC-QE-MS (UHPLC-Q-Exactive Orbitrap MS). QC as well as qualitative and quantitative analyses of metabolites, were conducted according to the methods described by Lv et al. (Lv et al., 2022). For mass spectrum data analysis, principal component analysis (PCA) and partial least squares discriminant analysis (PLS-DA) were applied. Differential accumulated metabolites (DAMs) among wheat varieties were identified using the PLS-DA model, applying VIP values greater than 1, log_2_foldchange (Log_2_FC) thresholds of ≥ 1 or ≤ -1, a *P*-value of less than 0.05, and examining ploidy changes of the peak area. DAMs were annotated based on the KEGG database, followed by enrichment pathway analysis.

### 2.5 Quantitative reverse transcription PCR

Gene expression levels were analyzed using AceQ qPCR SYBR Green Master Mix (Vazyme, Nanjing, China) according to the manufacturer’s instructions. Amplified primers for the target genes were designed using Premier 5. Primer specificity was accessed using the NCBI primer design tool (https://www.ncbi.nlm.nih.gov/tools/primer-blast/index.cgi?LINK_LOC=BlastHome). The relative transcription levels were calculated using the 2^−ΔΔCt^ method (Livak and Schmittgen, 2001).

### 2.6 Histochemical detection of ROS using 3,3’-diaminobenzidine staining

Wheat leaves were fully immersed in 2 mL of DAB staining solution (DAB 1 mg/mL, pH = 3.8), and a suitable length of inoculated wheat leaves was selected for staining. The samples were incubated overnight (8-12h) at room temperature in dark. For decolorizing the staining solution was removed, and the samples were placed in a boiling water bath until colorless. Subsequently, 5mL of fixing solution (composed of anhydrous ethanol, glacial acetic acid, glycerol 3:1:1 ratio) was added. After cooling to room temperature, the leaves were transferred to fresh deionized water and imaged using an optical microscope.

## 3 Results

### 3.1 Wheat line ‘Lr29NIL’ exhibited varying resistance to LR under different temperature conditions

**Figure 1:**
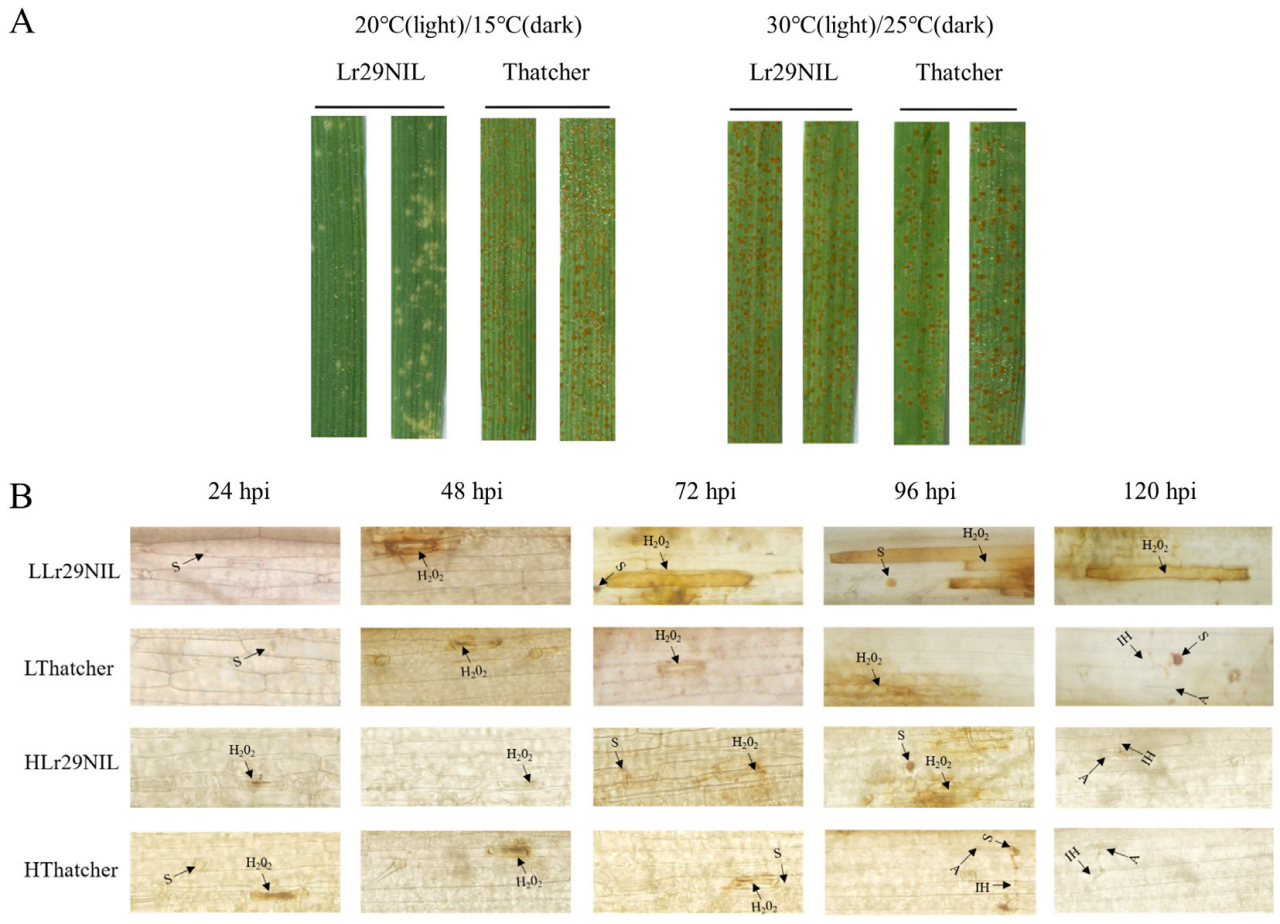
Reactions of ‘Lr29NIL’ and ‘Thatcher’ to *Pt* race D-3-2-182. **(A)** Infection types of ‘Lr29NIL’ and ‘Thatcher’ at low and high temperatures, observed at ~10 dpi. **(B)** DAB staining of ‘Lr29NIL’ and ‘Thatcher’ leaf tissues after *Pt* inoculation at different temperatures and hpi. S, spore; A, appressorium; IH, infection hyphae; L, Low temperature; H, High temperature.

In our studies, we found that under low temperature condition of 20°C during the day and 15°C at night, the ‘Lr29NIL’ seedlings scored ‘;’ IT (high resistance) to *Pt* race D-3-2-182, while ‘Thatcher’ scored ‘4’ IT (highly susceptible), according to the Stakman et al. (1962) scale (**Figure 1A**). However, when the plants were grown at high temperature condition of 30°C during the day and 25°C at night, both ‘Lr29NIL’ and ‘Thatcher’ exhibited highly susceptible phenotype (IT ‘4’) (**Figure 1A**). These results indicate that the resistance conferred by *Lr29* is influenced by environmental temperature.

### 3.2 Accumulation of ROS and plant disease resistance

To determine whether the resistance conferred by *Lr29* in ‘Lr29NIL’ correlates with ROS accumulation and whether this is affected by temperature variation, we performed DAB staining. Comparisons were made between ‘Lr29NIL’ and the susceptible control, ‘Thatcher’, under low-temperature (LLr29NIL vs LThatcher) and high-temperature conditions (HLr29NIL and HThatcher) (**Figure 1B**).

DAB staining revealed that, in the early stages of *Pt* infection, H_2_O_2_ was produced at the stomatal appressoria, indicating localized production of H_2_O_2_ at this site. Staining in ‘Thatcher’ showed that the generation of H_2_O_2_ may result from physical pressure on the stomata caused by the appressorium and invading mycelium. As the infection progressed, these sites in the ‘Lr29NIL’ began to accumulate ROS. Notably, a substantial accumulation of ROS was detected in mesophyll cells surrounding the infection sites of ‘Lr29NIL’ after 72 hpi, this phenomenon not observed in ‘Thatcher’ or HLr29NIL (**Figure 1B**). These findings were consistent with the phenotype results of inoculation.

### 3.3 Transcriptional analysis of wheat response to temperature change after inoculation with *Pt*

RNA-seq was performed on the ‘Lr29NIL’ and its backcross parent ‘Thatcher’, yielding a total of approximately 2,697 million clean reads, with an average of 112 million reads per sample. The overall sequencing error rate was 0.02%, while Q20 and Q30 values exceeded 97% and 93%, respectively. The average GC content was 53.23% (**Supplementary Table S1**). These results indicate high sequencing quality, ensuring the data is suitable for subsequent analyses. To standardize gene expression levels, FPKM was employed, and Pearson correlation coefficients demonstrated a high level of reproducibility in gene expression data among samples (**Supplementary Figure S1**).

**Figure 2:**
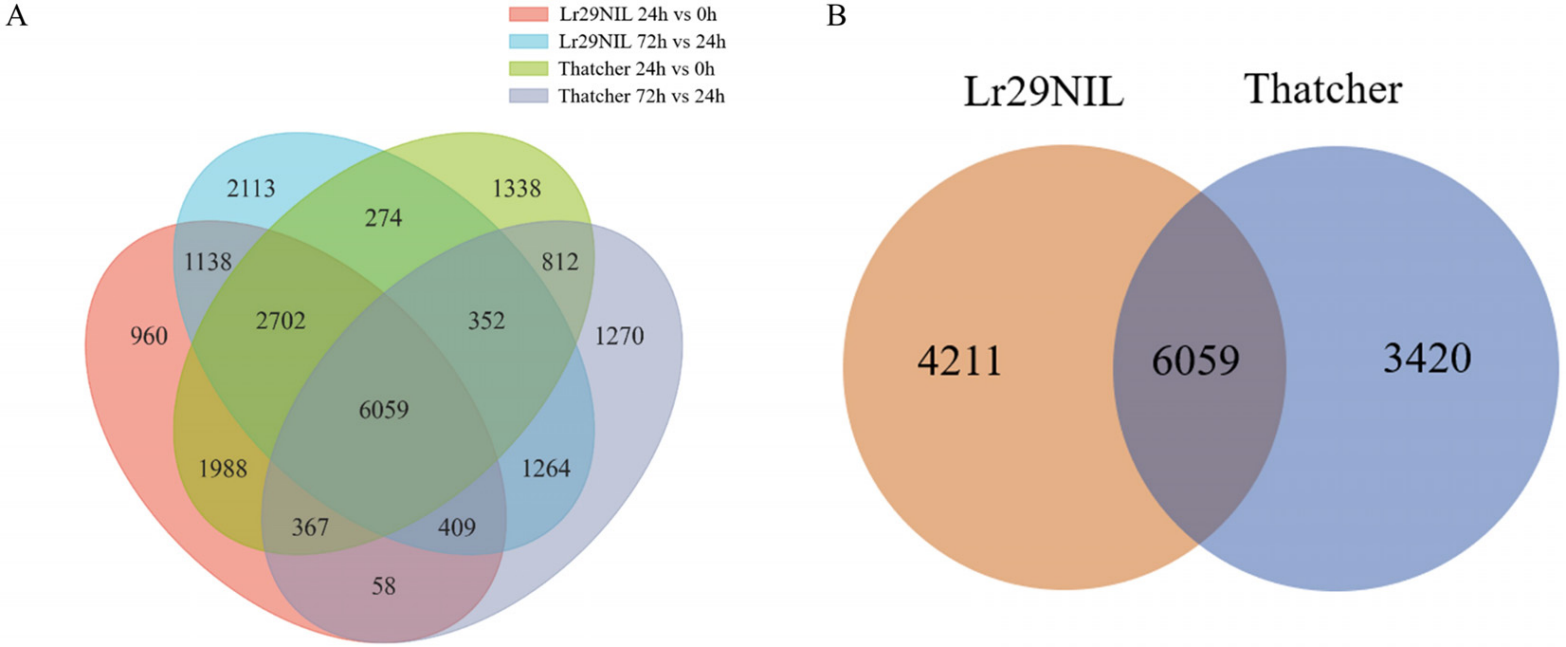
Analysis of DEGs under leaf rust infestation. **(A)** Numbers of DEGs identified in the comparisons between ‘Lr29NIL’ and ‘Thatcher’ under low temperature conditions. **(B)** Venn diagram showing the overlap of DEGs between ‘Lr29NIL’ and ‘Thatcher’ under low temperature conditions.

**Figure 3:**
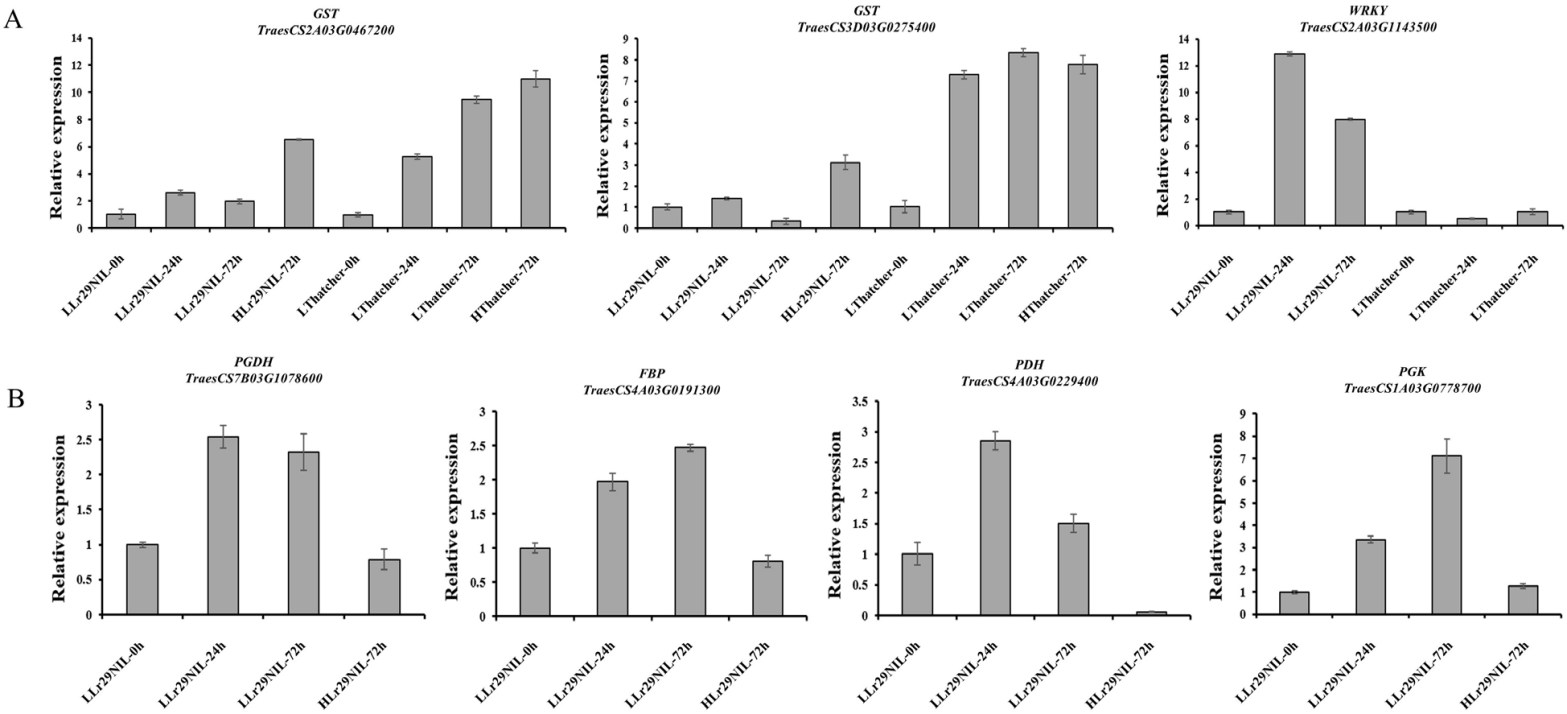
Validation of RNA-seq results by qRT-PCR. **(A)** Relative expression levels of selected genes in ‘Lr29NIL’ and ‘Thatcher’ at different time points post-inoculation. **(B)** Relative expression levels of selected genes in LLr29NIL and HLr29NIL at 72 hpi. L, Low temperature; H, High temperature.

The DEGs of ‘Lr29NIL’ and ‘Thatcher’ in low temperature was analyzed (LLr29NIL and LThatcher) at various hpi-s. In total, 21,104 DEGs were identified in LLr29NIL and LThatcher following infection with *Pt* at different time points (**Figure 2A**). In addition, 13,690 genes exhibited significant differential regulation between LLr29NIL and LThatcher, while 4,211 genes were specifically expressed in ‘Lr29NIL’ and 3,420 genes were specifically expressed in ‘Thatcher’ (**Figures 2A, B**). These findings indicate that *Pt* infection significantly alters gene expression, highlighting substantial differences between ‘Lr29NIL’ and ‘Thatcher’ wheat genotypes. To validate the RNA-seq data, 7 DEGs were further analyzed using qRT-PCR (**Supplementary Table S2**). As illustrated in **Figure 3**, the expression patterns corresponded closely with the transcriptome findings, thereby reinforcing the reliability of the RNA results.

When comparing DEGs at different time points, the fewest DEGs were identified at 0 hpi between LLr29NIL and LThatcher (**Supplementary Figure S2**). Of these DEGs, 2,069 were located on the chromosome 7E/7D translocation, while only 362 were mapped to other chromosomes (**Supplementary Figure S3**).

To determine the functions of the DEGs identified, we conducted GO analysis, which revealed enrichment of DEGs in three major functional categories: molecular function (MF), cellular component (CC), and biological process (BP). The top 30 enriched GO terms across various comparisons of DEGs are illustrated in **Supplementary Figures S4A–D**. Genes associated with oxidoreductase activity were the most abundant DEGs identified in the comparisons between LLr29NIL and LThatcher at both 24 and 72 hpi (**Supplementary Figures S4A, B**). In the 72 hpi comparison of ‘Lr29NIL’ and ‘Thatcher’ at high- and low-temperature conditions, all three GO categories (MF, CC, and BP) showed enrichment in DEGs related to amino acid and energy metabolism (**Supplementary Figures S4C, D**).

**Figure 4:**
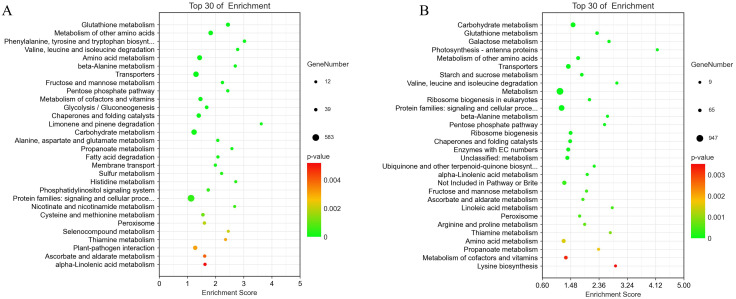
KEGG pathways analysis at different time points between ‘Lr29NIL’ and ‘Thatcher’ following infection with *Pt.* **(A)** Scatterplot of KEGG pathways of DEGs in LLr29NIL vs LThatcher at 24 hpi. **(B)** Scatterplot of KEGG pathways of DEGs in LLr29NIL vs LThatcher at 72 hpi.

**Figure 5:**
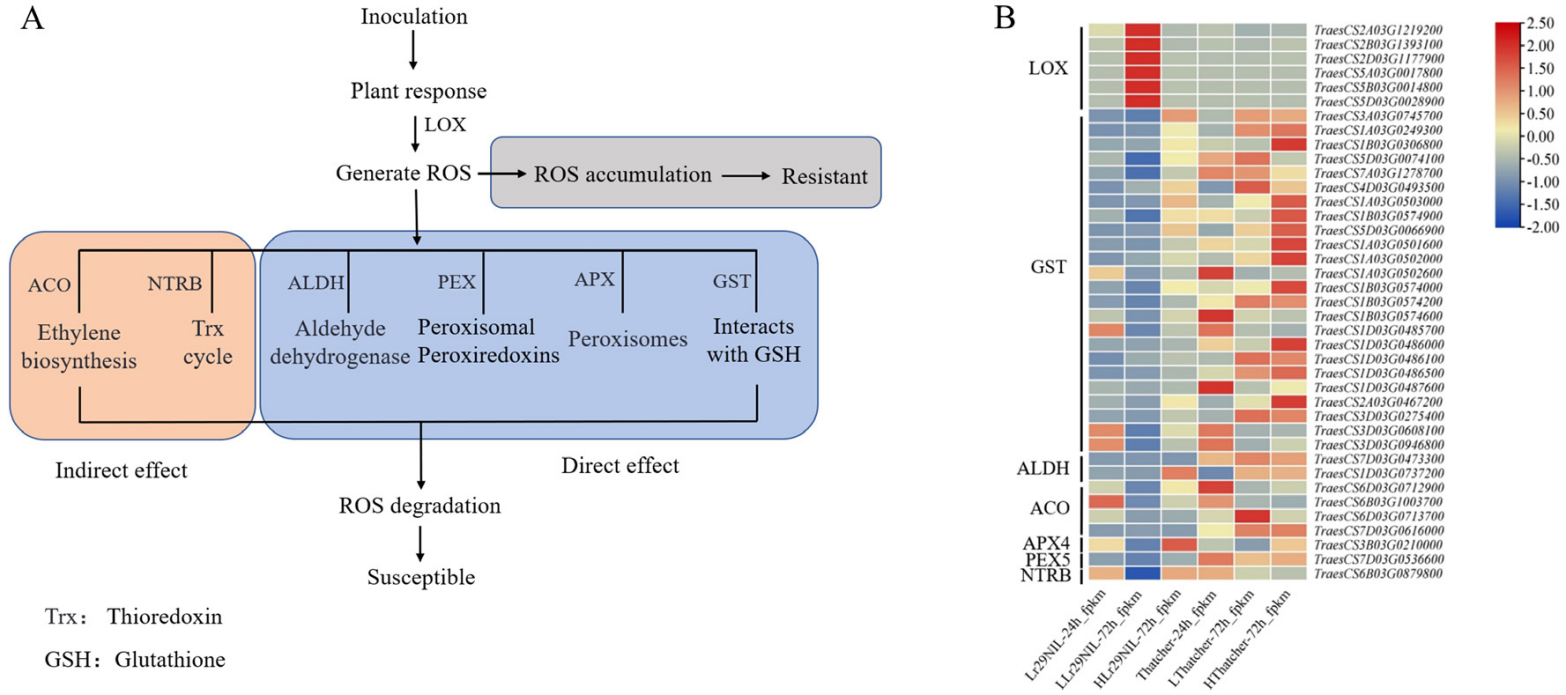
The transcriptomic analysis of the ROS metabolic pathway. **(A)** Overview of the ROS metabolic pathway. Indirect effect: Indirect involvement in ROS clearance. Direct effect: Directly involvement in ROS clearance. **(B)** Differential expression of the key genes involved in the ROS metabolic pathway. The heatmap scale ranges from -2 to +2.5 on a log_2_FC. L, Low temperature; H, High temperature.

To further explore the biological functions of DEGs, we performed a KEGG enrichment analysis. Between 24 and 72 hpi, the primary pathways enriched for ‘Lr29NIL’ and ‘Thatcher’ were amino acid metabolism (phenylalanine, tyrosine and tryptophan biosynthesis, valine, leucine and isoleucine degradation, alanine metabolism), ROS metabolism (glutathione metabolism, peroxisome), and pathogen-plant interactions (**Figure 4**). Our study identified pathways related to ROS, with LOX (lipoxygenase) genes linked to ROS production, while APX (ascorbate peroxidase), GST (glutathione S-transferase), ALDH (aldehyde dehydrogenase) and PEX (peroxisome) genes were associated with ROS elimination. Additionally, ACO (1-aminocyclopropane-1-carboxylate oxidase), NTRB (NADPH: thioredoxin reductase B) and GH3 (glutathione hydrolase 3) genes were implicated in indirect ROS elimination (**Figure 5**). We observed that the expression of genes related to ROS production increased in LLr29NIL but decreased in HLr29NIL, LThatcher, and HThatcher (**Supplementary Table S3**). Conversely, the genes associated with ROS clearance significantly decreased in the resistant materials, particularly 72 hpi, leading to ROS accumulation, this is consistent with the results in **Figure 1B**. In HLr29NIL, LThatcher and HThatcher, however, genes related to ROS clearance increased, resulting in effective ROS removal (**Supplementary Table S3**). Following inoculation, a KEGG pathways analysis was conducted comparing high and low temperatures materials at 72 hpi. The primary pathway affected by temperature in ‘Lr29NIL’ and ‘Thatcher’ was amino acid metabolism (**Supplementary Figures S5A, B**).

**Figure 6:**
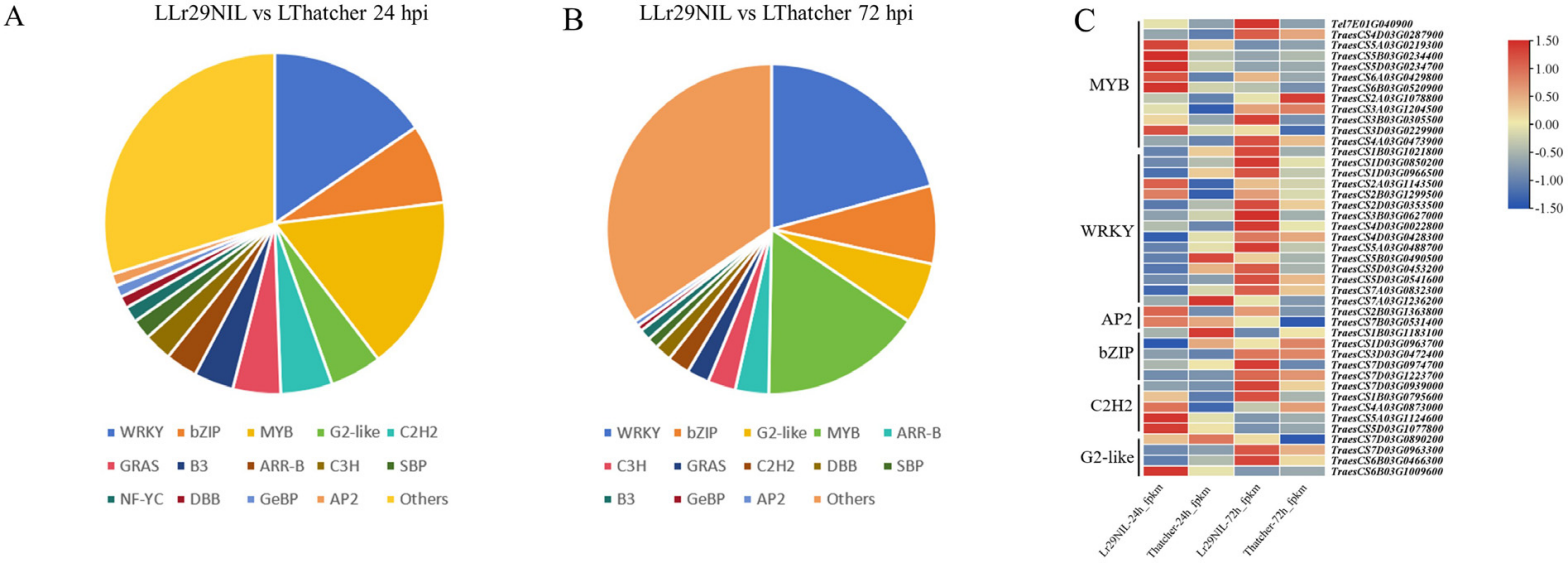
Differential expression of transcription factors (TFs) between LLr29NIL and LThatcher. **(A)** Pie chart showing the differentially expressed TFs in LLr29NIL and LThatcher at 24 hpi. **(B)** Pie charts illustrating the differential expression of LLr29NIL and LThatcher at 72 hpi. **(C)** Heatmaps of the expression patterns of MYB, WRKY, AP2, bZIP, C2H2 and G2 like between LLr29NIL and LThatcher at 24 and 72 hpi. L, Low temperature.

Given the crucial role transcription factors (TFs) play in regulating gene expression, we also examined their expression profiles at low temperature. In the paired comparisons (‘Lr29NIL’ vs ‘Thatcher’ at 24 hpi), we identified 265 differentially expressed TFs, which were categorized into 35 groups. In another paired comparison (LLr29NIL vs LThatcher at 72 hpi) we found 180 differentially expressed TFs across 31 categories (**Figures 6A, B**; **Supplementary Table S4**). These TFs belonged to five major families: MYB, WRKY, bZIP, G2-like, and C2H2, all of which play a vital role in plant defense against biotic stress. We further identified defense-related TFs specific to *Pt* infection from the ‘Lr29NIL’ and ‘Thatcher’ comparisons (**Figure 6C**; **Supplementary Table S5**). Although these candidate TFs were derived from the same families, they exhibited different expression patterns based on their transcription levels (**Supplementary Table S5**); some were highly expressed in ‘Lr29NIL’ at 24h, for example, WRKY (*TraesCS2A03G1143500*) and C2H2 (*TraesCS5D03G1077800*); while others peaked at different times after inoculation. In the case of LLr29NIL, the TFs from the MYB, WRKY, and C2H2 families exhibited higher transcript levels compared to those in LThatcher, indicating a positive regulation of wheat resistance to LR. Notably, the WRKY TFs *TraesCS2A03G1143500*, *TraesCS2B03G1299500* and *TraesCS4D03G0022800* were up-regulated in LLr29NIL, suggested that these TFs may play significant roles in enhancing wheat resistance to LR (**Supplementary Table S5**). This study highlights that these TFs could serve as key regulators of downstream genes associated with LR resistance, contributing to the observed differences in resistance between ‘Lr29NIL’ and ‘Thatcher’.

### 3.4 Metabolome involvement of wheat response to temperature change after inoculation with *Pt*

**Figure 7:**
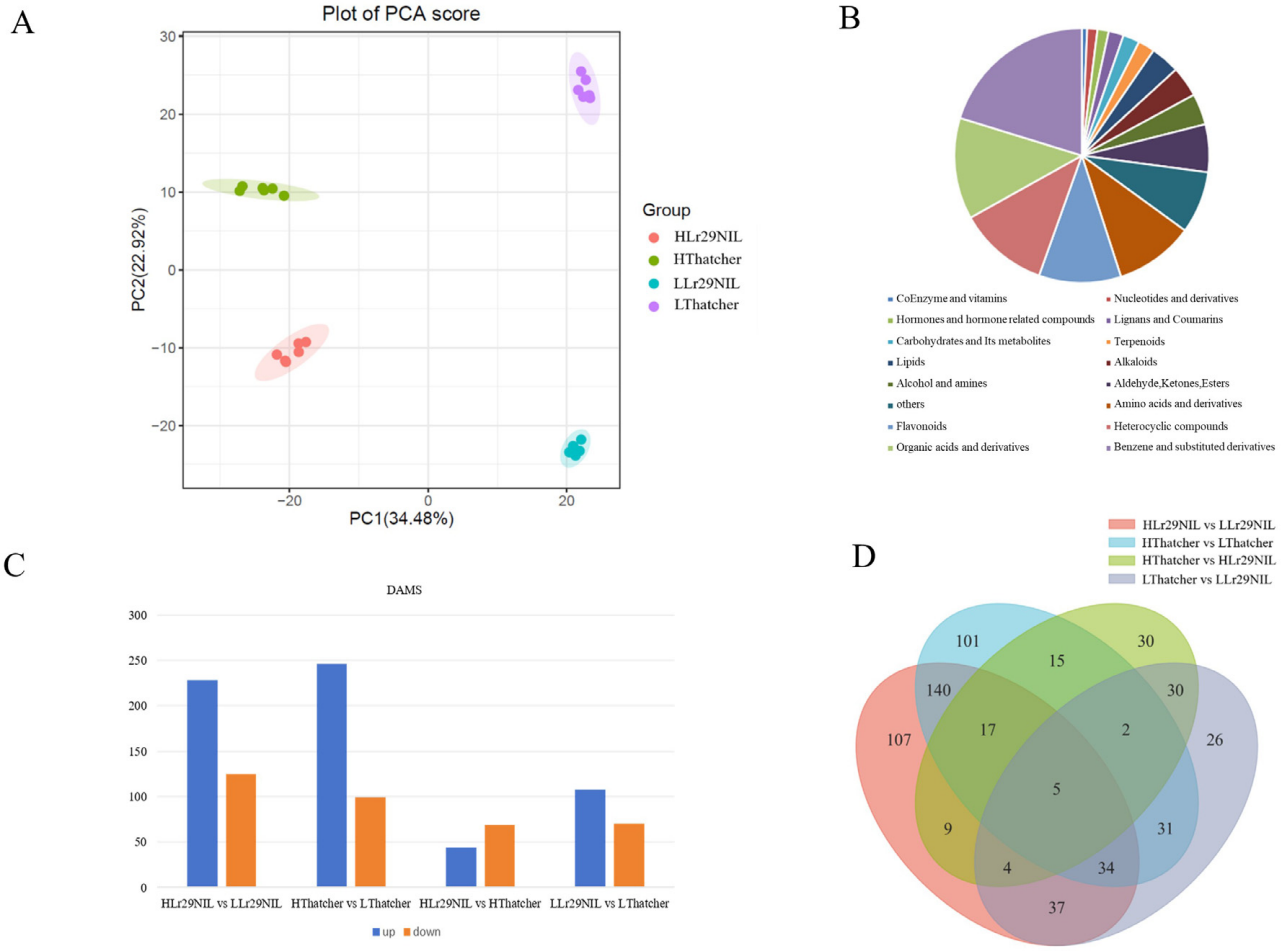
Analysis of DAMs under leaf rust infestation. **(A)** PCA score plot of mass spectrometry data for each sample group and quality control samples. **(B)** Pie chart showing the distribution of metabolites. **(C)** Numbers of upregulated and downregulated DAMs in different comparisons. **(D)** Venn diagram of DAMs across different comparisons. L, Low temperature; H, High temperature.

We utilized Pearson’s Correlation Coefficient (r) to evaluate the correlation of biological replicates; values closer to 1 indicate a stronger correlation between duplicate samples (**Supplementary Figure S6A**). The quantitative metabolites resulted from all samples were analyzed using PCA. PCA analysis highlighted significant differences between PC1 and PC2, with PC1 accounting for 34.48% and PC2 for 22.92% of the variance (**Figure 7A**). The PCA score and sample correlation diagrams revealed a r-value exceeding 0.8 across the four sample groups (LLr29NIL, HLr29NIL, LThatcher, HThatcher) at 72 hpi, indicating strong repeatability and high intragroup correlation (**Supplementary Figure S6B**).

A total of 1,467 metabolites were identified across four samples through qualitative and quantitative analyses (**Supplementary Table S6**). These metabolites were classified into 16 categories, which encompass five main types: benzene and its substituted derivatives, organic acids and their derivatives, heterocyclic compounds, flavonoids, and amino acids and their derivatives (**Figure 7B**). Using criteria of VIP > 1, *P*-value < 0.05, absolute log_2_FC ≥ 1, the DAMs were identified across various comparisons.

After 72 hpi, 169 metabolites were found to be differentially accumulated in the LLr29NIL vs. LThatcher comparison, comprising 108 upregulated and 61 downregulated DAMs (**Figure 7C**). The HLr29NIL vs. HThatcher comparison revealed 112 differentially altered metabolites, including 44 upregulated and 68 downregulated DAMs (**Figure 7C**). In addition, we identified 353 DAMs (228 upregulated and 125 downregulated) in the HLr29 NIL vs. LLr29NIL comparison, and 345 DAMs (246 upregulated and 99 downregulated) in the HThatcher vs. LThatcher comparison (**Figures 7C, D**). Overall, compared with the condition of inoculation, temperature change has a greater effect on the number of DAMs. At the same high temperature, DAMs expression between HLr29NIL and HThatcher were less pronounced. These findings suggest that plants respond to stress by enhancing the expression of specific metabolites.

To identify defense-related metabolites, we screened DAMs log_2_FC ≥ 2 in LLr29NIL compared to LThatcher. Detailed information is provided in **Table 1**. In comparison to ‘Thatcher’, Laricitrin 3-glucoside, a type of flavonoid, was identified as the most significantly up-accumulated metabolite, displaying a log_2_FC of 5.25. Notably, among of the top 13 up-accumulated metabolites, there are 9 flavonoids, while the remaining include 1 alkaloid, 1 cinnamate, 1 sugar acid, and 1 naphthoquinone compound. This suggests that the accumulation of flavonoids may play a crucial role in wheat’s resistance to LR.

**Table 1:** Significantly different metabolites in LLr29NIL and LThatcher at 72 hpi.

No.	Name	Formula	Log_2_(FC)	Category
meta0467	Laricitrin 3-gulcoside	C_22_H_22_O_13_	5.25	Flavonol glycoside
meta0429	Senecionine N-oxide	C_18_H_25_NO_6_	5.00	alkaloid
meta0735	Chrysoeriol	C_16_H_12_O_6_	4.45	Flavonoids
meta0729	Syringetin-3-o-glucoside	C_23_H_24_O_13_	2.97	Flavonol glycoside
meta0359	1-O-trans-Cinnamoy1-beta-D-glucopyranose	C_15_H_18_O_7_	2.35	Cinnamate esters
meta0659	Myricitrin	C_28_H_24_O_17_	3.24	Flavonol glycoside
meta0084	Rutin	C_27_H_30_O_16_	3.09	Flavonol glycoside
meta0152	Vitexin-2’’-O-glucoside	C_27_H_30_O_16_	2.82	Flavonol glycoside
meta0605	Meloside A	C_27_H_30_O_15_	2.59	Flavonol glycoside
meta0043	Vitexin	C_21_H_20_O_10_	2.23	Flavonol glycoside
meta0020	Cosmosiin	C_21_H_20_O_10_	2.13	Flavonol glycoside
meta0381	D-Gulono-1,4-lactone	C_6_H_10_O_6_	2.05	Saccharic acid
meta0443	Thelephantin E	C_33_H_24_O_8_	2.00	Naphthoquinones

**Figure 8:**
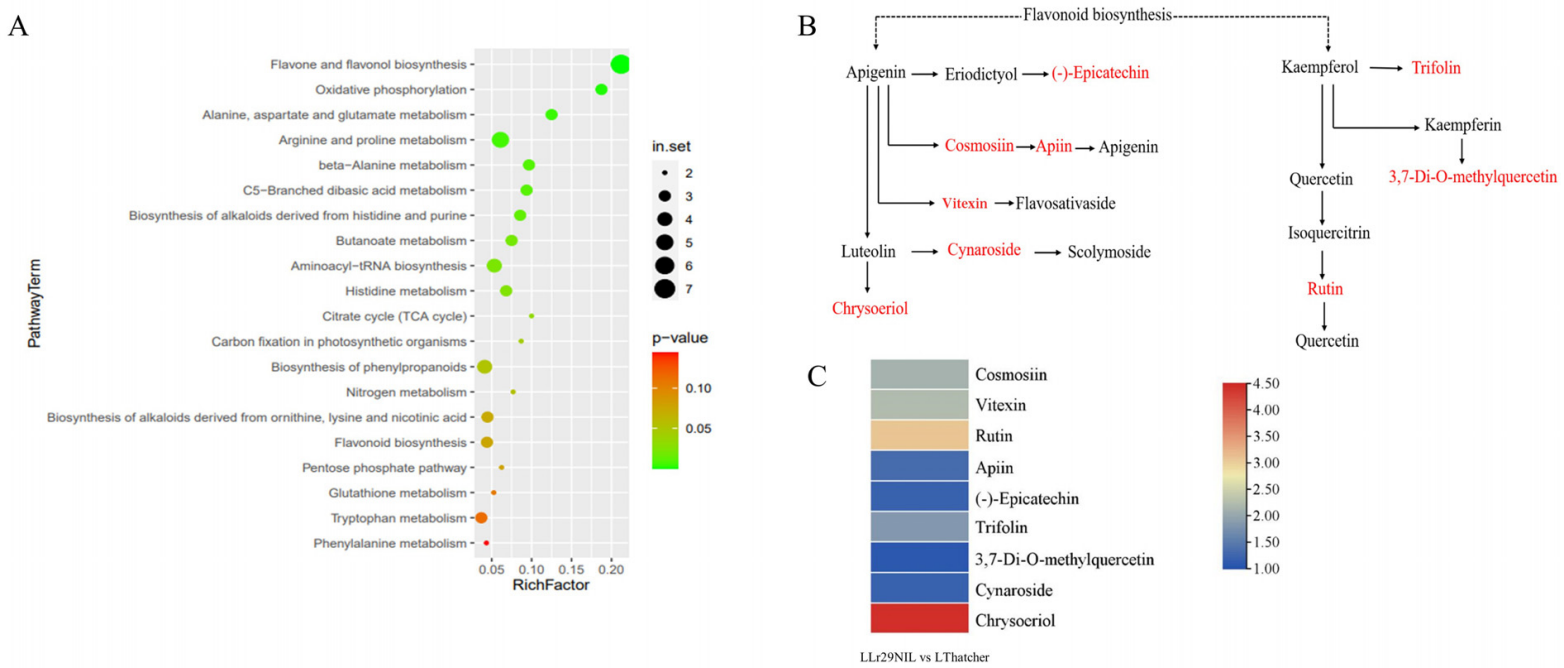
Metabolomic analysis of the flavonoid biosynthesis pathway. **(A)** Scatterplot of KEGG pathways for DEGs in ‘Lr29NIL’ vs ‘Thatcher’ at 72 hpi. **(B)** Flavonoid metabolic pathway. Key differentially accumulated metabolites in the wheat LLr29NIL vs LThatcher comparison at 72 hpi are highlighted in red. **(C)** Differential expression of key metabolites involved in the flavonoid biosynthesis pathway. The heatmap scale ranges from + 1.08 to +4.45 on a log_2_FC. L, Low temperature; H, High temperature.

We conducted KEGG annotation and enrichment analysis of DAMs to identify key metabolic pathways involved in wheat’s defense against LR. Several KEGG pathways were enriched, with the top 20 pathways listed in **Figure 8** and **Supplementary Figure S7**. The analysis highlighted that the DAMs are primarily associated with amino acid metabolism, particularly the shikimic acid metabolism pathway, which is essential for the synthesis and metabolism of aromatic amino acids such as phenylalanine, tyrosine and tryptophan (**Supplementary Figures S7A, B**). Our results indicate that the shikimic acid metabolic pathway plays a significant role in plant responses to elevated temperature.

In addition to amino acid metabolism, flavone and flavonol biosynthesis are the major differential pathways between ‘Lr29NIL’ and ‘Thatcher’ at low temperatures, highlighting the importance of flavonoids in LR resistance (**Figure 8**). However, under high temperature conditions, the differences in metabolites between ‘Lr29NIL’ and ‘Thatcher’ were minimal, and the number of enriched pathways was significantly lower (**Supplementary Figure S7C**). These findings suggest that, under high temperature conditions, the metabolite profiles of ‘Lr29NIL’ and ‘Thatcher’ do not differ significantly.

### 3.5 Integrated analysis of metabolomic and transcriptomic

**Figure 9:**
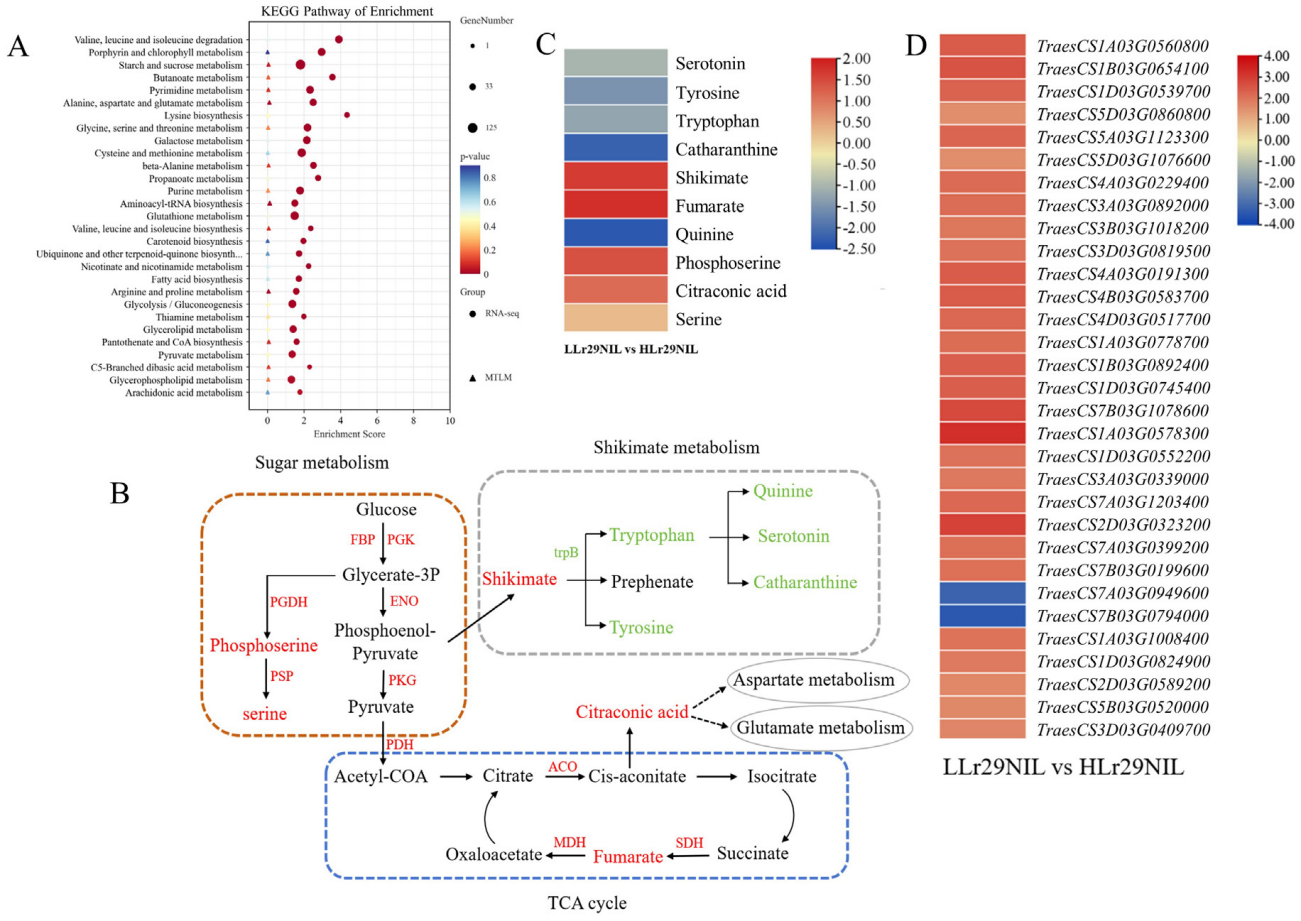
Correlation analysis of DEGs and DAMs in the comparison between LLr29NIL vs HLr29NIL at 72 hpi. **(A)** KEGG analysis of DEGs and DAMs that were enriched in the same pathway in the LLr29NIL vs HLr29NIL. **(B)** Pathways related to disease resistance and influenced by temperatures. Red and green colors indicate significant increases and decreases in abundance, respectively. **(C)** Differential expression of key metabolites involved in these pathways. The heatmap scale ranges from -2.35 to +2.32 on a log_2_FC. **(D)** Differential expression of key genes involved in these pathways. The heatmap scale ranges from -3.43 to +3.26 on a log_2_FC. L, Low temperature; H, High temperature.

To investigate the impact of rising temperature on LR resistance of ‘Lr29NIL’, we performed analysis of the DEGs and DAMs within KEGG pathways of the ‘Lr29NIL’ transcriptome and metabolome under high and low-temperature conditions over 72 hpi. Our findings indicated that energy metabolism (sugar metabolism, TCA cycle) and amino acid metabolism (shikimic acid metabolism) were the two pathways exhibiting the greatest differential enrichment (**Figure 9A**; **Supplementary Table S8**). Generally, variations in enzyme-related genes were consistently linked to changes in metabolite expression (**Supplementary Tables S9**, **S10**).

The results showed that genes and metabolites associated with glycolysis and the tricarboxylic acid cycle were up-regulated in LLr29NIL, while those related to shikimic acid metabolism were up-regulated following temperature increases (**Figures 9B–D**). These findings suggest that the energy metabolism pathway may be linked to *Lr29* resistance, whereas the shikimic acid pathway may be influenced by rising temperature. In LLr29NIL, the metabolites phosphoserine, serine, citrate, fumarate, and citraconic acid were found to be up-regulated, indicating a positive correlation with LR resistance. Accordingly, metabolites such as tryptophan, tryptophan, quinine, serotonin, and catharanthine in the shikimic acid metabolic pathway were up-regulated in HLr29NIL, mirroring the results observed in ‘Thatcher’ under both high and low temperature conditions. This suggest that these metabolites may also respond to temperature increases (**Supplementary Figure S8**).

## 4 Discussion

As LR is one of the most widespread and severe diseases of wheat, the understanding of mechanism underlying wheat’s defense against LR is essential. This study utilized ‘Lr29NIL’ carrying *Lr29* gene as a LR resistance material and the backcrossing parent ‘Thatcher’ as a susceptible control. Both materials were inoculated under low and high temperature conditions. We investigated the resistance mechanisms gene *Lr29*, as well as the impact of increased temperatures on the loss of efficiency of *Lr29* in the seedling stage, using transcriptomic and metabolome analyses.

### 4.1 Effect of ROS in the wheat response to *Pt* infection

During pathogen infection, the accumulation of ROS, specifically hydrogen peroxide (H_2_O_2_) and superoxide anion (O^2-^), has been demonstrated to influence plant resistance to disease (Wang et al., 2007; Orczyk et al., 2010). The initial response involves the production of ROS molecules, including hydrogen peroxide (H_2_O_2_) and superoxide anions (O_2_^−^) (Bolwell and Wojtaszek, 1997). These ROS molecules act as signaling agents and are essential for both plant growth and defense mechanisms (Levine et al., 1994; Dat et al., 2000). Studies have also shown that the WKS1 protein functions by inducing the accumulation of H_2_O_2_ and triggering programmed cell death (PCD), which contributes to stripe rust resistance (Gou et al., 2015).

It was shown that LOX generates bioactive peroxides by oxidizing polyunsaturated fatty acids such as linoleic acid and arachidonic acid (Feussner and Wasternack, 2022). Additionally, these products can participate in oxidative reactions that lead to the production of more ROS (Feussner et al., 2001). Consequently, in plants, increased LOX activity often correlates with elevated ROS levels, contributing to rapid responses to pathogen invasion and environmental stress (Chehab et al., 2007). During hypersensitive responses, glutathione plays a crucial role in regulating ROS accumulation (Zechmann, 2020). Previous transcriptomic studies have supported the induction of specific glutathione S-transferase (GST) groups during the early phase of plant-pathogen interactions (Gullner et al., 2018). In sugarcane, a rapid accumulation of ROS was observed following infection with the tobacco mosaic virus (TMV). Compared to resistant genotypes, susceptible genotypes exhibited a significant increase in GST activity to scavenge ROS, indicating that GST is an important marker for plant pathogen attacks (Akbar et al., 2020). In addition to GST, we found that genes such as APX, ALDH, PEX, ACO, and NTRB are also involved in ROS clearance, either directly or indirectly (**Figures 5A, B**). In our study, LLr29NIL at 72 hpi, the expression of ROS scavenging genes was lower than that in HLr29NIL, LThatcher and HThatcher, while the transcription level of LOX was significantly upregulated. Furthermore, our findings suggest that LOX is crucial for enhancing wheat resistance to LR by producing ROS (**Figure 5B**; **Supplementary Table S3**). The interaction between wheat and LR mediated by *Lr29* also led to a substantial accumulation of H_2_O_2_ in the stomatal and mesophyll cells at 72 hpi after inoculation (**Figure 1B**), indicating that the resistance gene *Lr29* can regulate disease resistance by influencing ROS levels.

### 4.2 Effect of flavonoids in the wheat response to *Pt* infection

Flavonoid compounds, a major class of plant secondary metabolites, are known to provide protection against biotic stresses and play a significant role in plant–microbe interactions (Mierziak et al., 2014; Bag et al., 2022). In our study, the flavonoid biosynthesis pathway was found to be enriched in DAMs between the resistant LLr29NIL and the susceptible LThatcher, which are well-established as a typical metabolic pathway related to pathogen defense (**Figure 8A**). This biosynthesis pathway has also been shown to be associated with resistance to various pathogens, including *Fusarium zanthoxyli* in *Zanthoxylum bungeanum*, *Sphaerotheca fuliginea* in cucumber, *Gymnosporangium yamadai* in apple leaves, and powdery mildew in wheat (Hoseinzadeh et al., 2020; Li et al., 2021; Zhang et al., 2021; Xu et al., 2023). Metabolomics analysis was applied to study the mechanisms underlying resistance to spot blotch in Yunnan Iron Shell wheat, revealing that flavonoids play a significant role in conferring resistance to powdery mildew (Zhang et al., 2022; Xu et al., 2023). Our study focused on flavonoids biosynthesis pathway, particularly on two important flavonoids, apigenin and kaempferol, which are known to contribute to disease resistance by protecting plants from pathogen invasion (**Figure 8B**). Moreover, in our study, several metabolites associated with flavonoid biosynthesis were expressed at higher level in ‘Lr29NIL’ compared to ‘Thatcher’ in this study (**Figure 8C**). Ullah et al. (2017) also found that similar metabolites, flavan-3-ols, were effective in chemical defense against rust infection in black poplar trees (*Populus nigra*).

Other metabolites in this pathway, such as cosmosiin, apiin, vitexin, cynaroside and chrysoeriol can also activate plant defense mechanisms (**Supplementary Table S7**) by inducing the expression of pathogenesis-related (PR) proteins, thus enhancing the plant’s resistance to pathogenic bacteria. Additionally, these metabolites can mitigate the risk of infection by inhibiting the growth and reproduction of pathogenic bacteria (Mamadalieva et al., 2011; Hartmann et al., 2018; Bangar et al., 2023; Boro et al., 2023). For instance, a study showed that tomato plants treated with chrysoeriol exhibited increased resistant to *Botrytis cinerea* following infection (Remali et al., 2022). Recent research has also demonstrated the role of rutin in defending against various bacterial infections. For example, external application of rutin can inhibit the proliferation of *Xanthomonas oryzae* pv. *Oryzae*, thereby improving rice resistance (Yang et al., 2016). In tomatoes, rutin, a secondary metabolite flavonoid, is crucial for enhancing resistance to *B. cinerealis* (Zhao et al., 2023).

### 4.3 Effect of temperature change on metabolic pathway of *Lr29* material

To analyze the reasons for *Lr29* susceptibility to high temperatures, a combined KEGG analysis was conducted on the DEGs and metabolites of ‘Lr29NIL’ exposed to *Pt* for 72 hpi at both high and low temperatures. The analysis revealed significant enrichment in pathways related to amino acid metabolism and energy metabolism (**Figure 9A**). These findings suggest that the loss of resistance of *Lr29* at elevated temperatures may be linked to these metabolic pathways.

Fumaric acid, an important organic acid and intermediate in the citric acid cycle, not only participates in energy metabolism but also plays a crucial role in disease resistance. Plants can release fumaric acid to inhibit pathogen colonization and survival, thereby enhancing their resistance to fungal infections (Khanna et al., 2019; Gurtler et al., 2020). In tomato plants, the application of fumaric acid has been shown to improve resistance to blight by enhancing the activity of antioxidant enzymes and reducing the invasion and spread of pathogens through the activation of defense gene expression (Singh et al., 2017).

Citraconic acid, a byproduct of the citric acid cycle, acts as a catalyst in the metabolism of aspartate and glutamate and regulates the synthesis of downstream amino acids (Li et al., 2023). Studies have demonstrated that citraconic acid can bolster the resistance of *Arabidopsis* to multiple pathogens (McFadden and Purohit, 1977; Li et al., 2023). It enhances the antioxidant capacity of plants by modulating metabolic pathways, activating the expression of defense genes, and improving overall disease resistance (McFadden and Purohit, 1977; Li et al., 2023).

In our study, we observed that following the increase in temperature, metabolites such as serine, citraconic acid, fumarate, shikimic acid and related genes were downregulated in HLr29NIL (**Supplementary Tables S9A**, **S10**). This downregulation may explain the observed loss of resistance in ‘Lr29NIL’ as temperature rises.

Furthermore, the increased expression of shikimic acid pathway-related metabolites in both ‘Lr29NIL’ and ‘Thatcher’ following a rise in temperature suggests a relationship between these metabolites and temperature (**Supplementary Figures S7A, B**). Exposure to various stresses induces changes in the shikimate acid metabolism (Tzin and Galili, 2010; Less and Galili, 2008). The aromatic amino acids (AAAs), specifically L-tyrosine, L-phenylalanine, and L-tryptophan, are primarily synthesized in the plastids via the shikimate pathway (Tzin and Galili, 2010). Tryptophan is a crucial essential amino acid in plants, playing a key role in the biosynthesis of a wide array of secondary metabolites. Compounds produced from the tryptophan metabolic pathway, such as 5-hydroxytryptamine, indoleacetic acid (IAA), and various alkaloids (including quinine and vinblastine), are vital for plant stress resistance. Serotonin, for example, regulates the levels of osmotic substances (such as proline, soluble sugars, and betaine) in plants, helping to maintain intracellular water balance and enhances stress resistance (Less and Galili, 2008). Additionally, quinine and vinblastine improve plant stress resistance by modulating the content of antioxidant systems and osmoregulatory substances (Arnao and Hernández-Ruiz, 2014). As the temperature increased in HLr29NIL and HThatcher (**Supplementary Table S9B**), the levels of tryptophan, tyrosine, serotonin, and alkaloids such as quinine and vinblastine were found to be elevated, indicating that these metabolites play a significant role in the plant’s response to rising temperatures.

## 5 Conclusion

This study investigated the resistance mechanism of the *Lr29* gene and its loss of function at high temperatures. Transcriptome analysis revealed that the accumulation of ROS plays a key role in *Lr29*-mediated resistance to LR. Specifically, ROS levels were elevated in ‘Lr29NIL’, with an increased expression of genes involved in ROS generation, such as LOX, and a decrease in the expression of genes associated with ROS detoxification, including GST, APX, and ACO. As temperature increases, genes and metabolites associated with glycolysis and the tricarboxylic acid (TCA) cycle were downregulated, while those related to the shikimic acid pathway were upregulated. In summary, *Lr29* confers resistance to LR by regulating the accumulation and removal of ROS. However, the loss of resistance under elevated temperatures during the seedling stage is a complex biological process, warranting further investigation.

## Data Availability

The original contributions presented in the study are publicly available. This data can be found at the National Center for Biotechnology Information (NCBI) using accession number PRJNA1221348.
