# Supplementary material for: Integrated transcriptome and metabolome analyses reveals the mechanisms of function loss of Lr29 leaf rust resistance gene at high temperatures in wheat
Source: Front Plant Sci. 2025 Feb 26;16:1537921. doi: 10.3389/fpls.2025.1537921 (PMC11897511; doi:10.3389/fpls.2025.1537921)

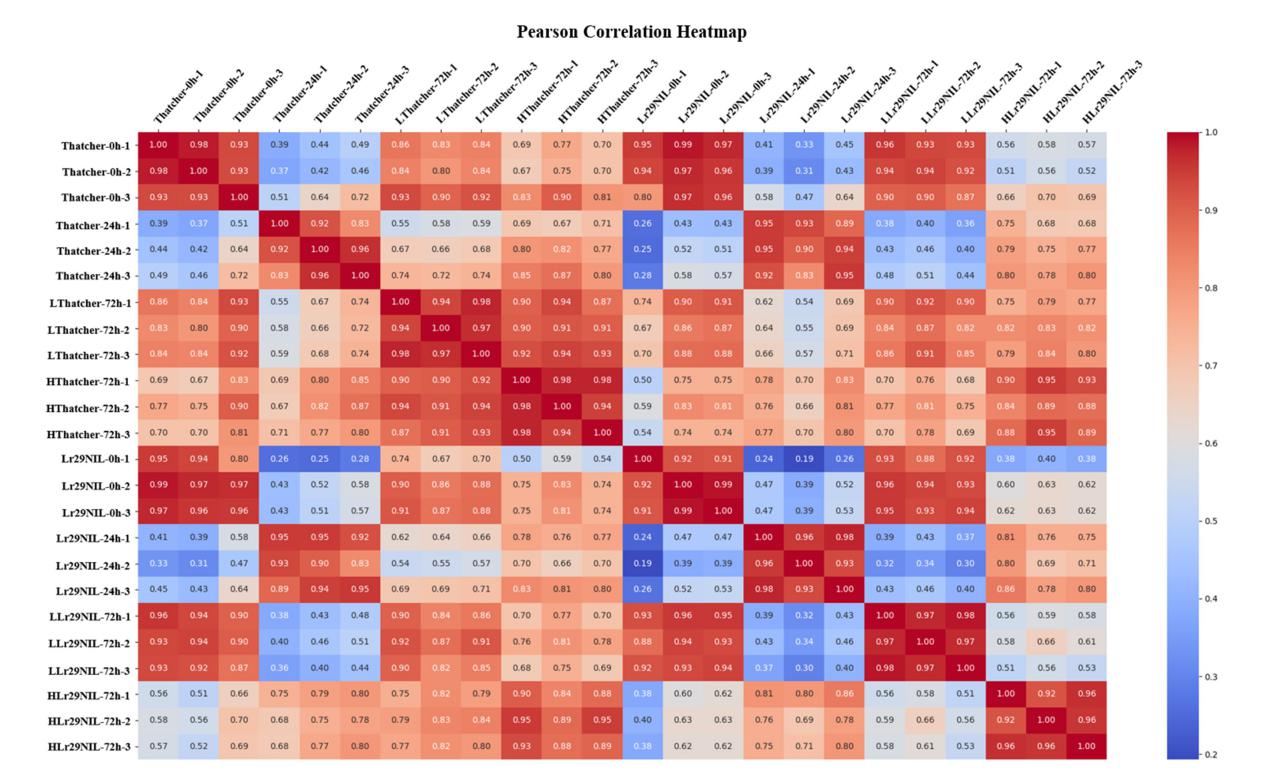


**Fig S1.** Diagram of correlation between samples. Note: L: Low temperature, H: High temperature.


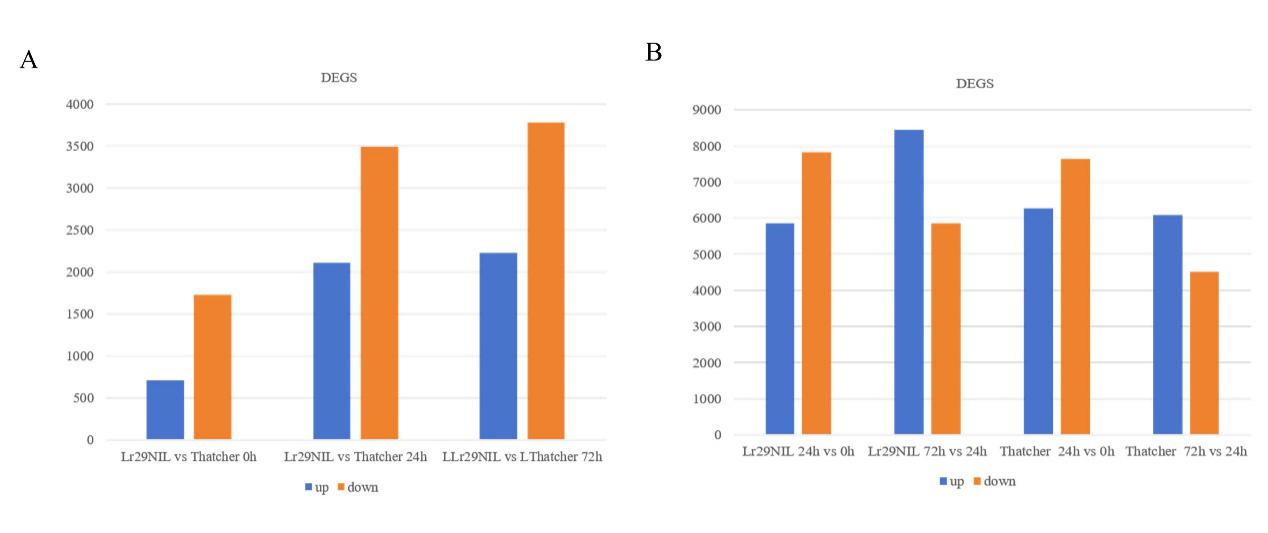


**Fig S2.** DEGs between the groups. (A) DEGs type at different time points between ‘Lr29NIL’ and ‘Thatcher’ following infection with *Pt*. (B) The DEGs between the same materials at different time points. Note: L: Low temperature, H: High temperature.


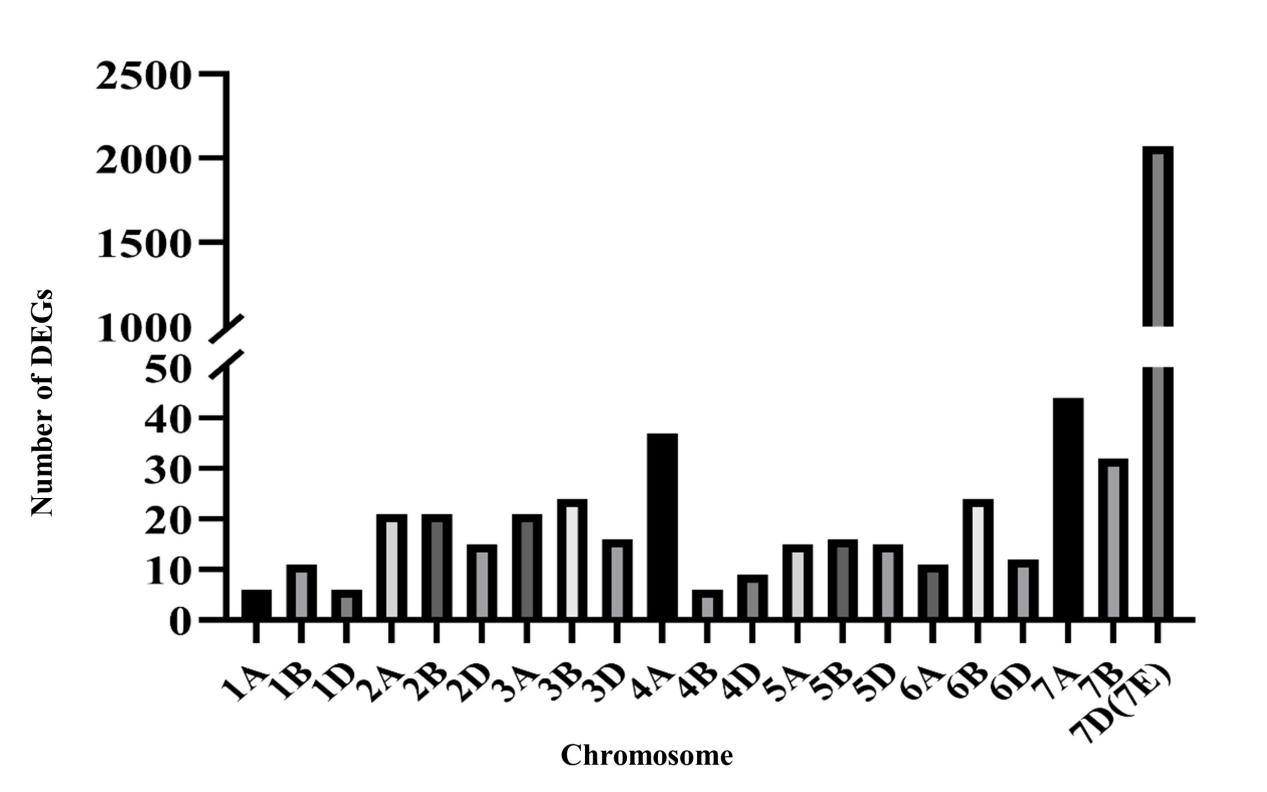


**Fig S3.** Number of differentially expressed genes (DEGs) in alien Tel chromosome and Chinese Spring (CS) genome between ‘Lr29NIL’ and ‘Thatcher’ at 0h. The alien Tel chromosome was 7E.


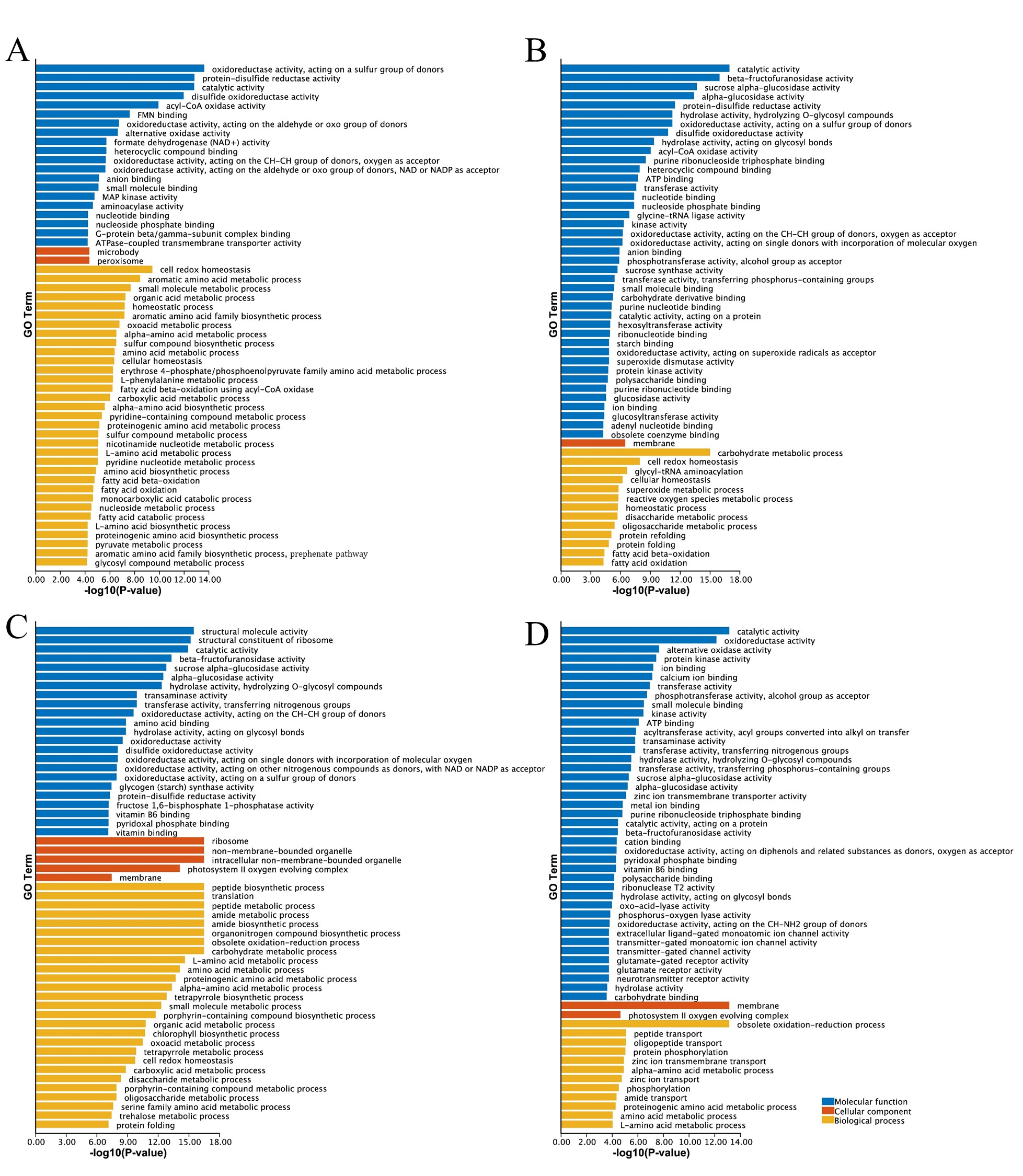


**Fig S4.** GO classification of transcriptomic data of LLr29NIL and LThatcher infected with *Pt.* (A) GO classification of DEGs in LLr29NIL vs LThatcher at 24 hpi. (B) GO classification of DEGs in LLr29NIL vs LThatcher at 72 hpi. (C) GO classification of DEGs in LLr29NIL vs HLr29NIL at 72 hpi. (D) GO classification of DEGs in LThatcher vs HThatcher at 72 hpi. Note: L: Low temperature, H: High temperature.


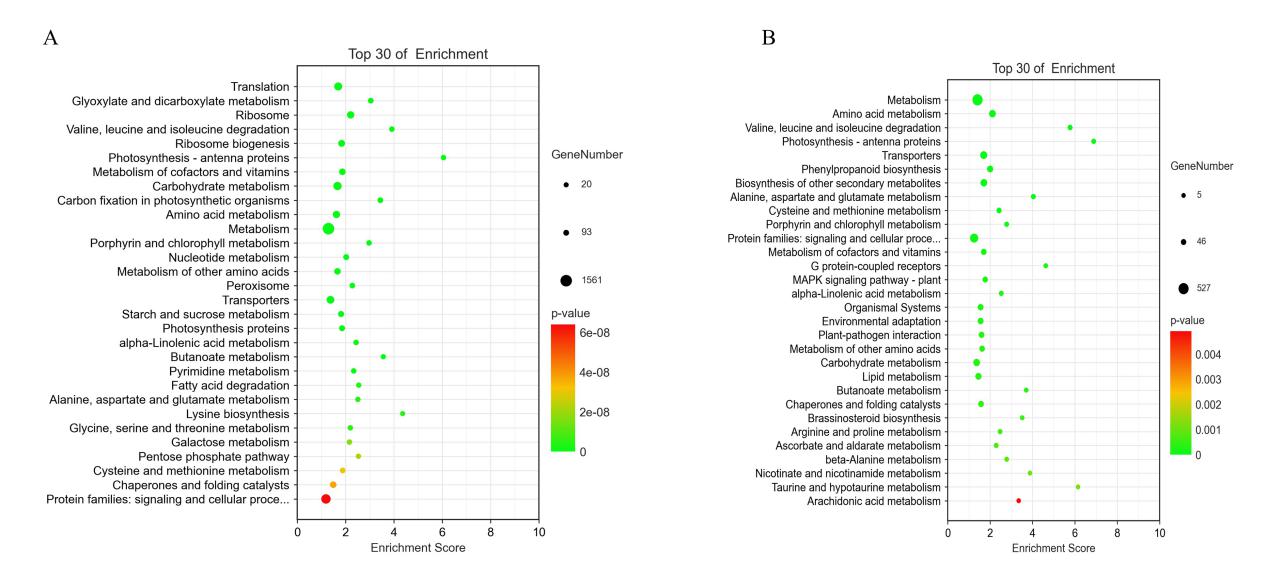


**Fig S5.** KEGG pathways analysis at same materials between high and low temperature following infection with *Pt.* (A) Scatterplot of KEGG pathways of DEGs in HLr29NIL vs LLr29NIL at 72 hpi. (B) Scatterplot of KEGG pathways of DEGs in HThatcher vs LThatcher at 72 hpi. Note: L: Low temperature, H: High temperature.


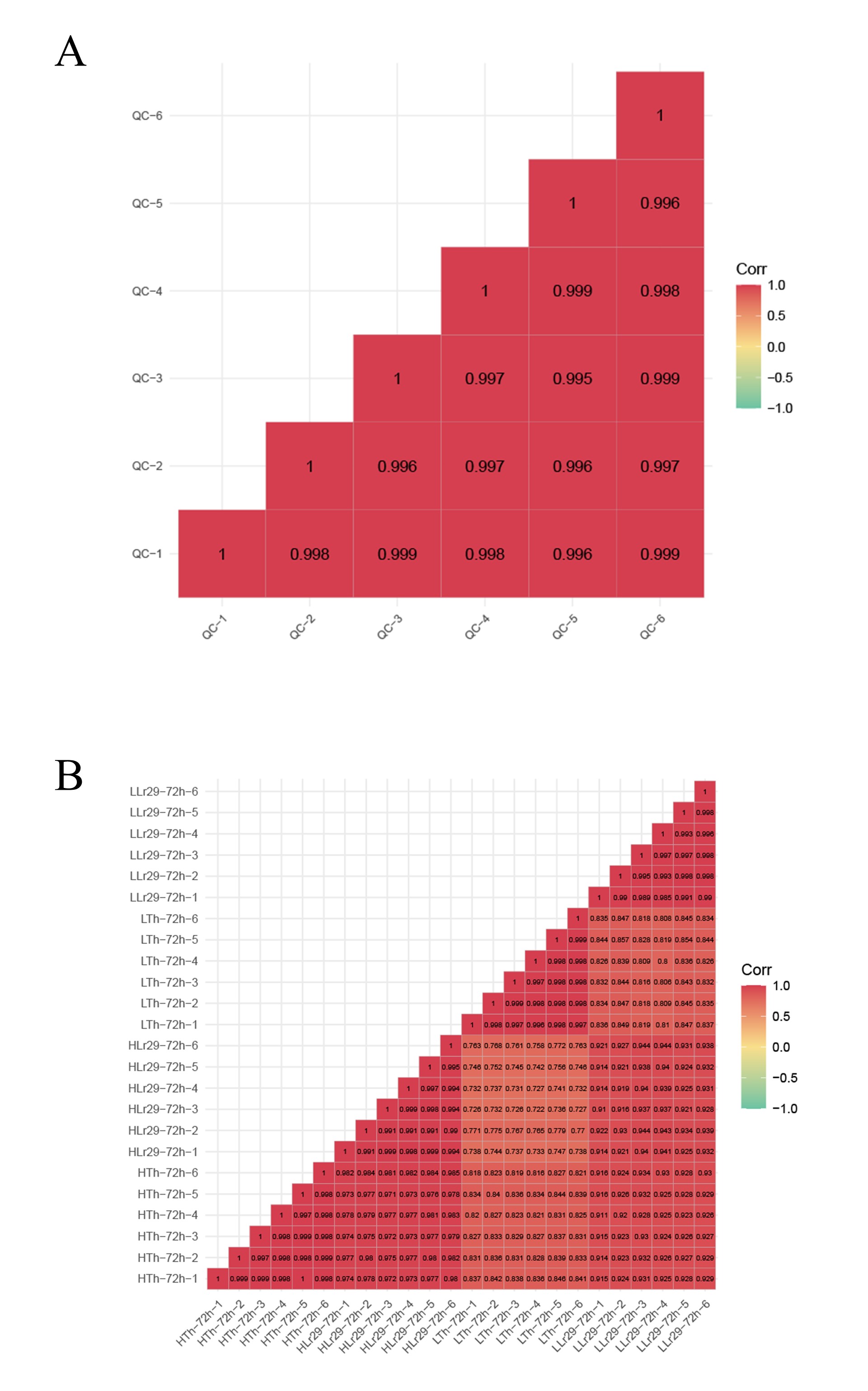


**Fig S6.** (A) QC diagram of correlation between samples. (B) Diagram of correlation between samples. Note: L: Low temperature, H: High temperature.

**Fig S7.** KEGG pathways analysis the DAMs of ‘Lr29NIL’ and ‘Thatcher’ at different temperature following infection with *Pt.* (A) Scatterplot of KEGG pathways of DAMs in LLr29NIL vs HLr29NIL at 72 hpi. (B) Scatterplot of KEGG pathways of DAMs in LThatcher vs HThatcher at 72 hpi. (C) Scatterplot of KEGG pathways of DAMs in HLr29NIL vs HThatcher at 72 hpi. Note: L: Low temperature, H: High temperature.


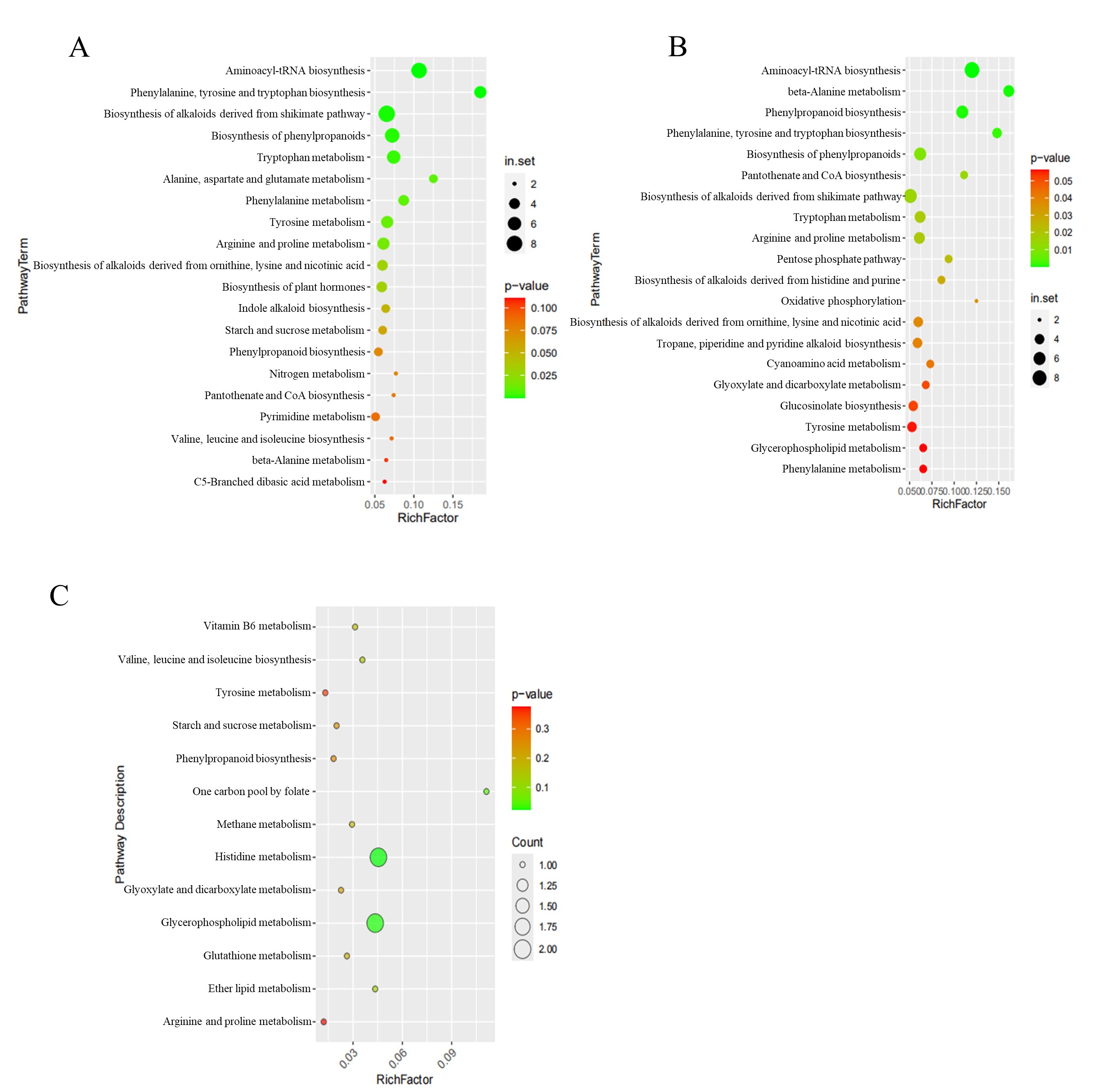


**Fig S8.** Differential expression of the key metabolites involved in Shikimic acid metabolic pathway at LLr29NIL vs HLr29NIL and LThatcher vs HThatcher. The heatmap scale ranges from -2.35 to +2.32 on a log_2_FC.


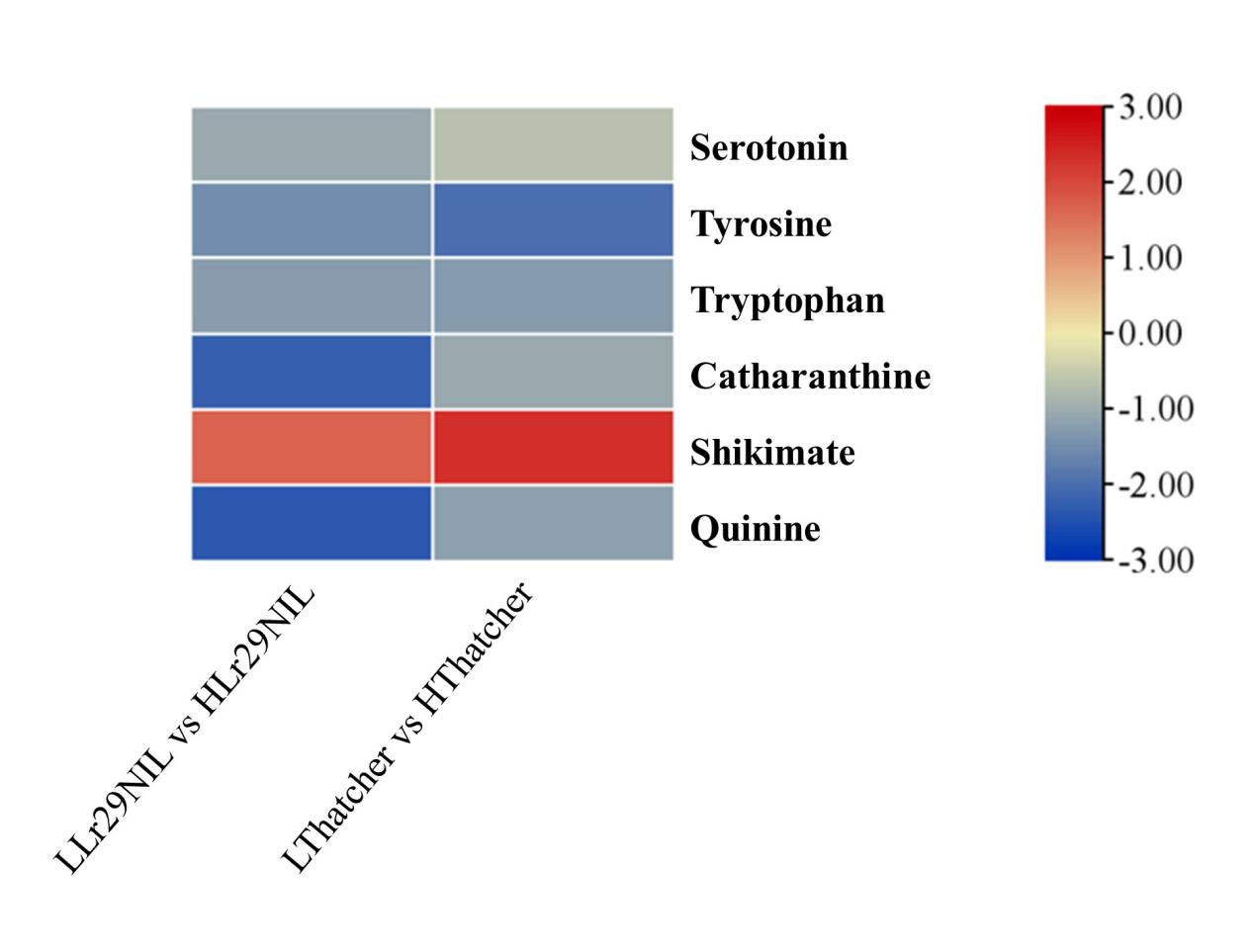

Supplement: Supplementary file 1 [file DataSheet1.docx]
